# Supplementary material for: Mental Fatigue, But Not other Fatigue Characteristics, as a Candidate Feature of Obsessive Compulsive Personality Disorder in Patients with Anxiety and Mood Disorders—An Exploratory Study
Source: Int J Environ Res Public Health. 2020 Nov 3;17(21):8132. doi: 10.3390/ijerph17218132 (PMC7662240; doi:10.3390/ijerph17218132)
Supplement: Supplementary file 1 [file ijerph-17-08132-s001.pdf]

Supplementary Table S1. Medication use of study participants, comparing the sub-groups with and without obsessive compulsive personality disorder (OCPD)*.

**Medications**	**Control group**	**OCPD group**	
	N (%)	N (%)	p
No medication	12 (18.5)	5 (25.0)	0.524
SSRI only	11 (16.9)	3 (15.0)	0.840
SSRI + BZD	9 (13.9)	4 (20.0)	0.506
SSRI+BZD+Mirtazapine	3 (4.6)	2 (10.0)	0.373
SSRI + antipsychotics	3 (4.6)	1 (5.0)	0.944
Antipsychotic only	3 (4.6)	0 (0.0)	0.331
SSRI + antipsychotics + BZD	3 (4.6)	0 (0.0)	0.331
Mirtazapine +Antipsychotics	3 (4.6)	0 (0.0)	0.331
SSRI + Agomelatine	3 (4.6)	0 (0.0)	0.331
Mirtazapine only	2 (3.1)	0 (0.0)	0.430
Agomelatine	2 (3.1)	0 (0.0)	0.430
BZD only	2 (3.1)	0 (0.0)	0.430
SSRI + Mirtazapine	1 (1.5)	0 (0.0)	0.578
Agomelatine + Tianeptine + BZD	1 (1.5)	0 (0.0)	0.578
SSRI+SNRI+BZD	1 (1.5)	0 (0.0)	0.578
Agomelatine+Antipsychotics	1 (1.5)	0 (0.0)	0.578
Agomelatine + BZD	0 (0.0)	1 (5.0)	0.073
BZD + Antipsychotics	1 (1.5)	0 (0.0)	0.578
SSRI + antipsychotics + BZD + Other (zolpidem)	1 (1.5)	0 (0.0)	0.578
Mirtazapine + Agomelatine + BZD + Antipsychotics	1 (1.5)	0 (0.0)	0.578
Mirtazapine + Bupropion + BZD	1 (1.5)	0 (0.0)	0.578
Mirtazapine + BZD	1 (1.5)	0 (0.0)	0.578
Mirtazapine + Anti-epileptic	0 (0.0)	1 (5.0)	0.073
SSRI + BZD + Anti-epileptic	0 (0.0)	1 (5.0)	0.073
Anti-epileptic+ Antipsychotic	0 (0.0)	1 (5.0)	0.073
SNRI only	0 (0.0)	1 (5.0)	0.073
Total	65	20	

Note: * defined operationally using the Compulsive Personality Assessment Scale. Values are given in numbers (%). p-value calculated with Fisher's Exact test for categorical variables. SSRI - selective serotonin reuptake inhibitors, BZD – benzodiazepines, SNRI - Serotonin and norepinephrine reuptake inhibitors
